# Supplementary figures and images for: Oncogenic and Tumor Suppressor Functions for Lymphoid Enhancer Factor 1 in E2a-/- T Acute Lymphoblastic Leukemia
Source: Front Immunol. 2022 Mar 18;13:845488. doi: 10.3389/fimmu.2022.845488 (PMC8971981; doi:10.3389/fimmu.2022.845488)

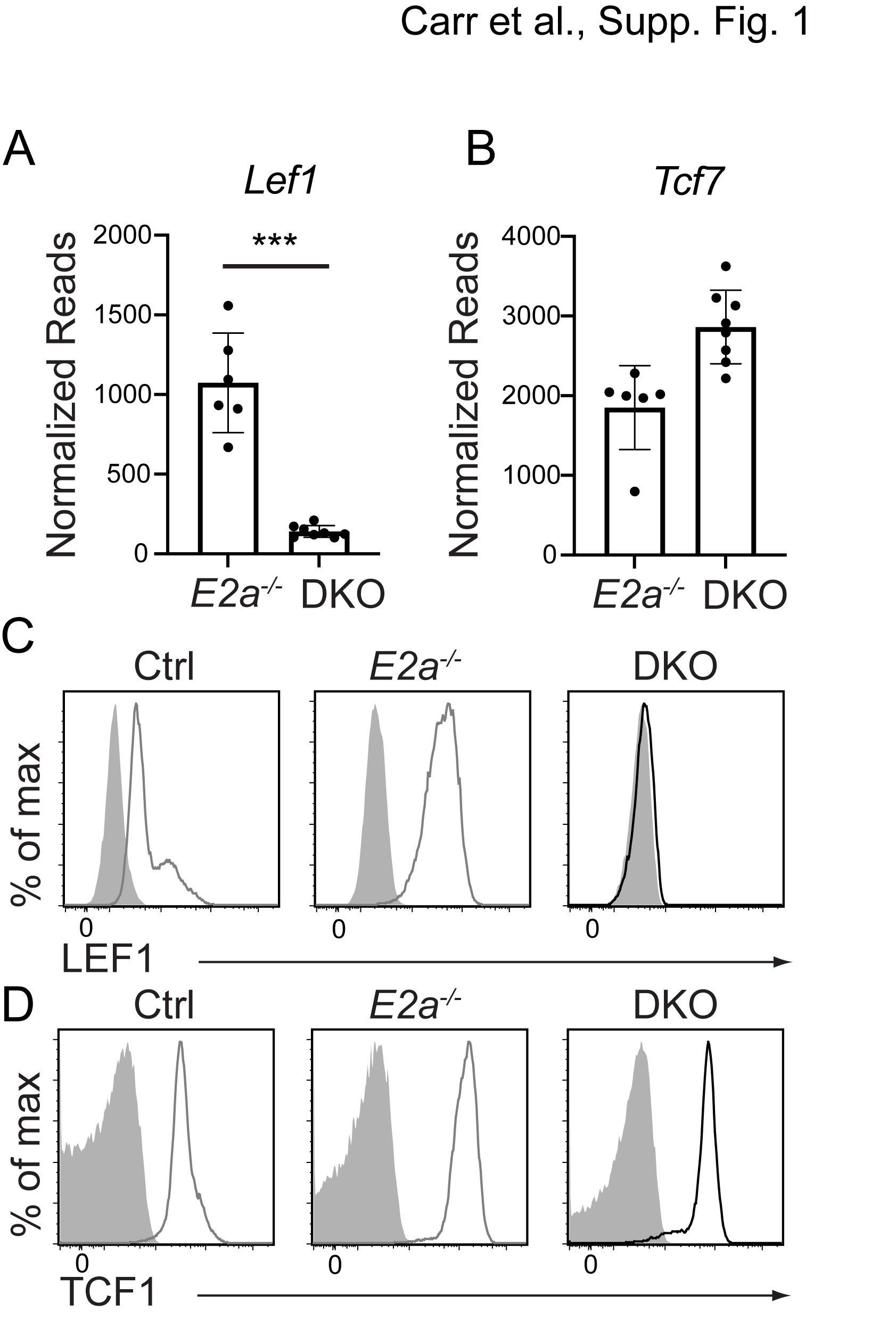

Supplement: Supplementary Figure 1 — Expression of LEF1 and TCF1 in E2a-/- and DKO leukemias. Normalized Reads for Lef1 (A) and Tcf7 (B) from RNA-sequencing data using RNA isolated from E2a-/- or DKO leukemia lines. Each dot represents the normalized reads from on line. Flow cytometry for (C) LEF1 and (D) TCF1 in Ctrl thymocytes (left panels) or an E2a-/- (middle panels) or DKO (right panels) leukemia. The shaded histogram is isotype control staining. ***p < 0.005. [file Image_1.tif]
